# Supplementary material for: Alleviation of Tris(2-chloroethyl) Phosphate Toxicity on the Marine Rotifer Brachionus plicatilis by Polystyrene Microplastics: Features and Molecular Evidence
Source: Int J Mol Sci. 2022 Apr 29;23(9):4934. doi: 10.3390/ijms23094934 (PMC9102492; doi:10.3390/ijms23094934)
Supplement: Supplementary file 1 [file ijms-23-04934-s001.zip › ijms-1677437-supplementary.pdf]

Supplementary Information

Alleviation of tris(2-chloroethyl) phosphate toxicity on the marine rotifer *Brachionus plicatilis* by polystyrene microplastics: features and molecular evidence

Wenqian Ma[1], Zijie Sun[1], Xin Zhang[1], Xuexi Tang[1,2,*],Xinxin Zhang[1,2,*]

Table S1. The ANOVA test among fluorescence intensity value of ingestion at different concentrations of MPs with size of 0.1 μm and 1 μm

		df	*F*	*p*
0.1 μm		3	1396.144	0.000
1 μm		3	285.877	0.000
p-values		20 μg/L	200 μg/L	2000 μg/L
0.1 μm	Control	<0.001	<0.001	<0.001
	20 μg/L		<0.001	<0.001
	200 μg/L			<0.001
1 μm	Control	<0.001	<0.001	<0.001
	20 μg/L		<0.001	<0.001
	200 μg/L			0.002

Table S2. The ANOVA test among life-history traits test at different concentrations of MPs with size of 0.1 μm and 1 μm

			df	*F*	*p*
0.1 μm	Average life span		3	0.017	0.997
	Total number of offsprings		3	0.096	0.960
1 μm	Average life span		3	6.644	0.015
	Total number of offsprings		3	0.361	0.783
	p-values		20 μg/L	200 μg/L	2000 μg/L
0.1 μm	Average life span	Control	0.894	1.000	0.864
		20 μg/L		0.894	0.970
		200 μg/L			0.864
	Total number of offsprings	Control	0.943	0.642	0.943
		20 μg/L		0.694	1.000
		200 μg/L			0.694
1 μm	Average life span	Control	0.783	0.004	0.067

	20 μg/L		0.007	0.104
	200 μg/L			0.109
	Control	0.611	0.574	0.963
Total number of offsprings	20 μg/L		0.326	0.604
	200 μg/L			0.628

Table S3. The ANOVA test among the population growth test of combined exposure environment-related concentration (EC) and high concentration (HC).

			df	*F*	*p*
EC		Tp	3	0.637	0.612
		K	3	2.773	0.111
HC		Tp	3	2.773	0.111
		K	3	0.361	0.783
	p-values		MPs-20 μg/L	TCEP-100 μg/L	MPs+TCEP
EC	Tp	Control	0.207	0.509	0.437
		MPs-20 μg/L		0.514	0.598
		TCEP-100 μg/L			0.901
	K	Control	0.130	0.024	0.071
		MPs-20 μg/L		0.311	0.702
		TCEP-100 μg/L			0.513
	p-values		MPs-2000 μg/L	TCEP-65 mg/L	MPs+TCEP
HC	Tp	Control	0.617	0.953	0.040
		MPs-2000 μg/L		0.557	0.036
		TCEP-65 mg/L			0.090
	K	Control	0.262	0.008	0.350
		MPs-2000 μg/L		0.030	0.928
		TCEP-65 mg/L			0.037

Table S4. The ANOVA test among the fluorescence intensity values of ROS and MDA levels of combined exposure concentration.

	df	*F*	*p*
ROS	3	12.871	0.000
MDA	3	4.589	0.054

p-values		MPs-2000 μg/L	TCEP-65 mg/L	MPs+TCEP
ROS	Control	＜0.001	＜0.001	＜0.001
	MPs-2000 μg/L		0.779	0.379
	TCEP-65 mg/L			0.546
MDA	Control	0.776	0.027	0.489
	MPs-2000 μg/L		0.018	0.329
	TCEP-65 mg/L			0.052

Table S5. Preprocessing results of sequencing data quality

Sample	RawReads	CleanReads	Q30	GC
Control -1	51.31M	50.61M	94.49%	39.23%
Control -2	45.55M	45.08M	95.11%	39.10%
Control -3	48.51M	47.89M	94.60%	39.41%
1 μm MPs-1	45.84M	45.17M	93.09%	39.53%
1 μm MPs-2	48.28M	47.60M	93.17%	39.01%
1 μm MPs-3	49.63M	48.93M	93.20%	39.63%
TCEP-1	49.59M	48.91M	93.37%	39.59%
TCEP-2	51.20M	50.62M	95.06%	39.72%
TCEP-3	47.63M	46.91M	92.93%	39.39%
1 μmMPs+TCEP-1	47.59M	46.98M	93.49%	39.52%
1 μmMPs+TCEP-2	51.27M	50.53M	93.14%	39.12%
1 μmMPs+TCEP-3	49.32M	48.69M	93.42%	39.49%

Table S6. Splicing result

Term	Unigene
All	24269
>=500bp	17417
>=1000bp	10961
N50	1963
Total_Length	32014050
Max_Length	31651
Min_Length	301
Average_Length	1319.13

Table S7. The number of unigene annotations in each database

Anno_Database	Annotated_Number
NR	12202(50.28 %)
Swissprot	10094(41.59 %)
KEGG	4121(16.98 %)
KOG	9111(37.54 %)
eggNOG	10900(44.91 %)
GO	9393(38.70 %)

Pfam	10565(43.53 %)

Table S8. The ANOVA test among the MXR activity of combined exposure concentration.

	df	*F*	*p*
Rhodamine B	3	3.740	0.060
Calcein AM	3	3.740	0.060
GST	3	31.394	0.000

p-values		MPs-2000 μg/L	TCEP-65 mg/L	MPs+TCEP
Rhodamine B	Control	0.361	0.700	0.028
	MPs-2000 μg/L		0.208	0.127
	TCEP-65 mg/L			0.015
Calcein AM	Control	0.036	0.001	0.001
	MPs-2000 μg/L		0.053	0.020
	TCEP-65 mg/L			0.555
GST	Control	$<$0.001	0.001	$<$0.001
	MPs-2000 μg/L		0.030	0.425
	TCEP-65 mg/L			0.092

Table S9. Primer_list

Gene Symbol	Forward primer(5->3)	Reverse primer(5->3)
EF1α	TGGCACATCACAAGCCGAT	GATACCGGCTTCGAATTCACC
TRINITY_DN6461_c0_g2_i1_1	GCTCAGATTAAAGAGTGGGT	TTTGGACTTGATACTCATGGT
TRINITY_DN4173_c0_g1_i1_1	GATCCATCCCAGCTTCCT	GCTCATAAATGCAGAGATGGTT
TRINITY_DN4922_c0_g1_i1_2	ACTTTTTGGACATTTACACCTG	CATGGGCTGGATGTGGAA
TRINITY_DN8383_c0_g1_i3_1	TGGCACATCACAAGCCGAT	GATACCGGCTTCGAATTCACC
TRINITY_DN7937_c0_g1_i1_2	CTGACTCTCTTATCAGTCCAG	ATTCCATTCCATTGTACAGGC
TRINITY_DN1210_c0_g1_i1_2	AGATTTTTACGGGACATGTGG	CCTTCTCCTAGCATACATAAGT

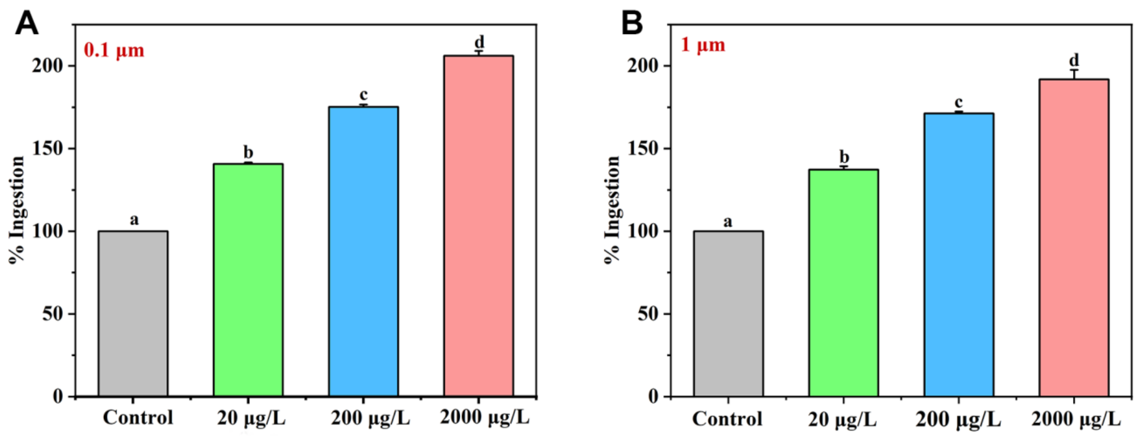

Figure S1. Quantification of fluorescence intensity of polystyrene microbeads (20, 200, and 2,000 µg/L) occurred in the rotifers after ingestion. Different lowercase above columns indicates significant differences ($p < 0.05$).

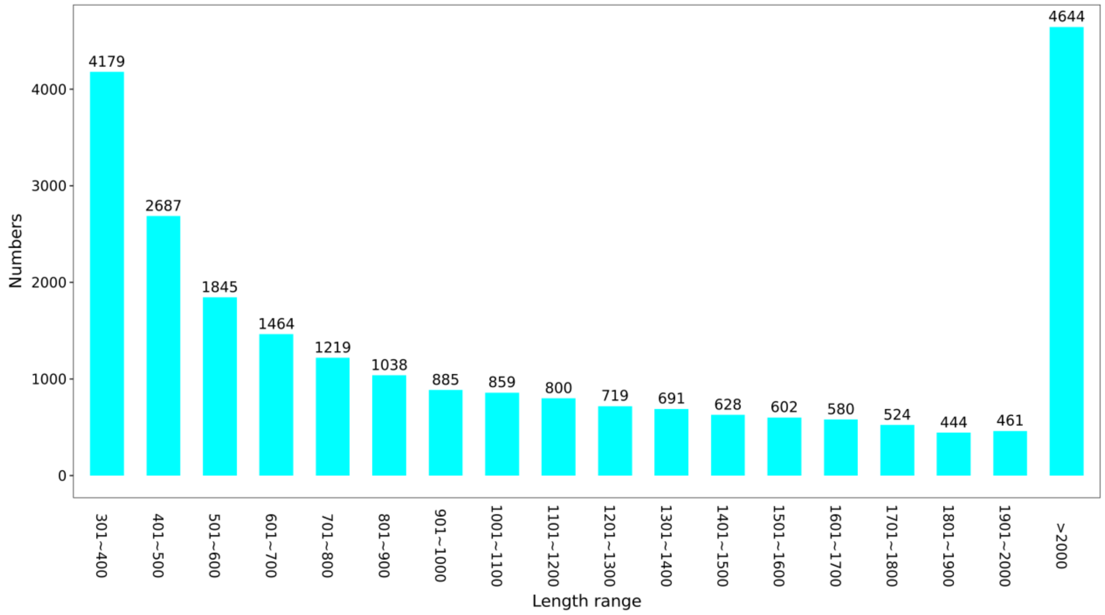

Figure S2. The distribution of sequence length.

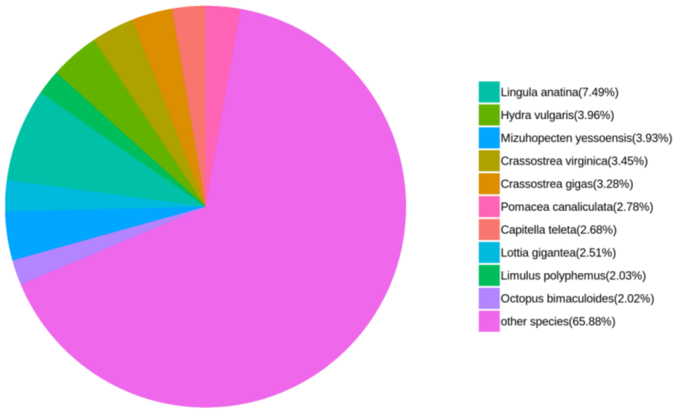

Figure S3. The distribution of top10 species in NR database.

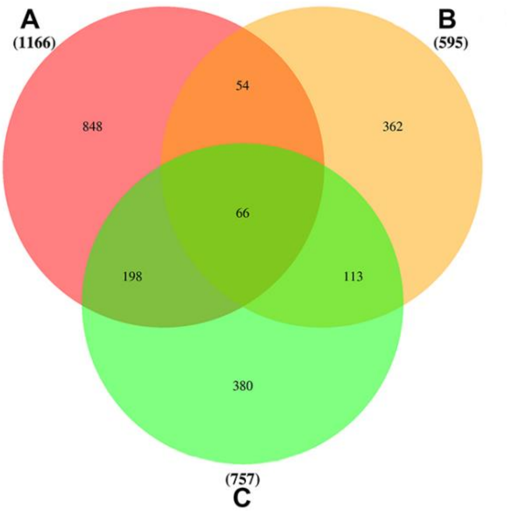

Figure S4. Venn diagram of differentially expressed genes in *B. plicatilis* after co-exposure to TCEP and 1 μm-MPs. (**A**) 1 μm-MPs vs Control; (**B**) TCEP vs Control; (**C**) 1 μm-MPs+TCEP vs Control.

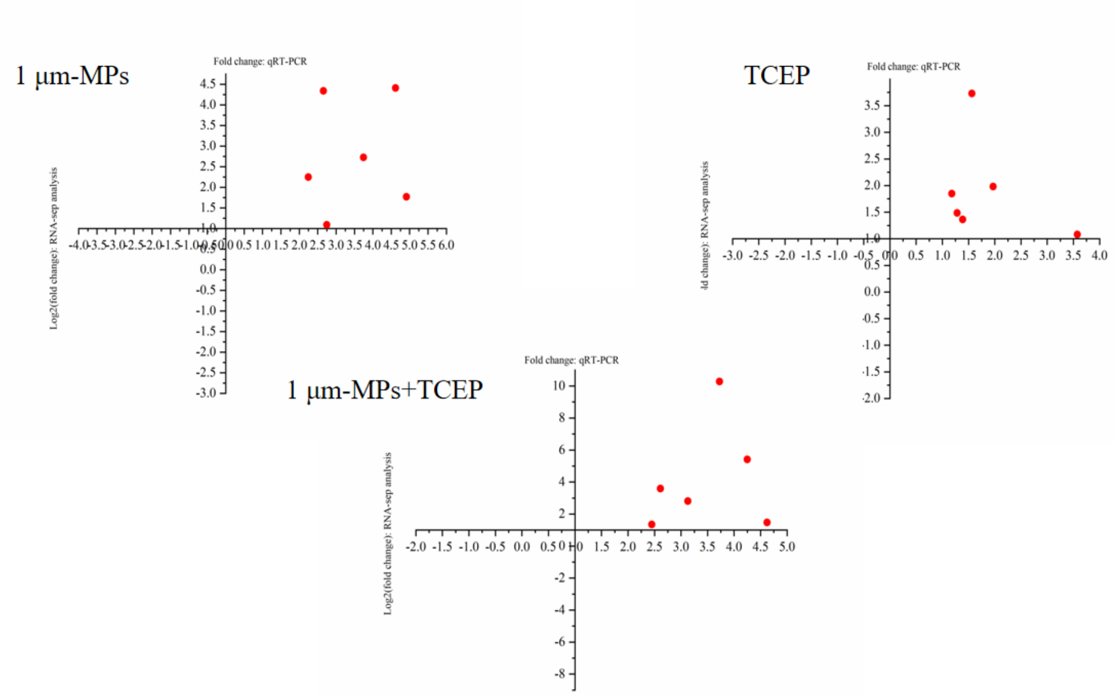

Figure S5. Comparison of fold change in gene expression between RNA-Seq analysis and qRT-PCR results.

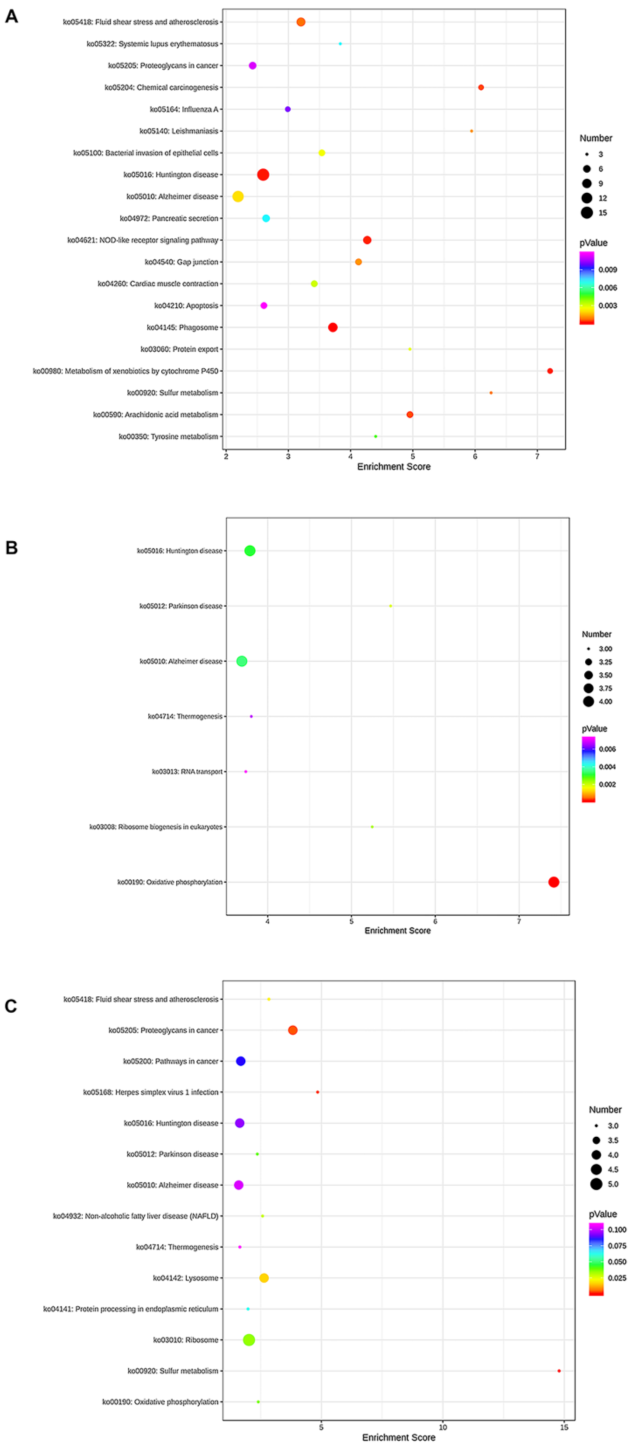

Figure S6. The top20 KEGG pathways that express significantly regulated gene enrichment. (**A**) 1 µm-MPs; (**B**) TCEP; (**C**) 1 µm-MPs+TCEP.

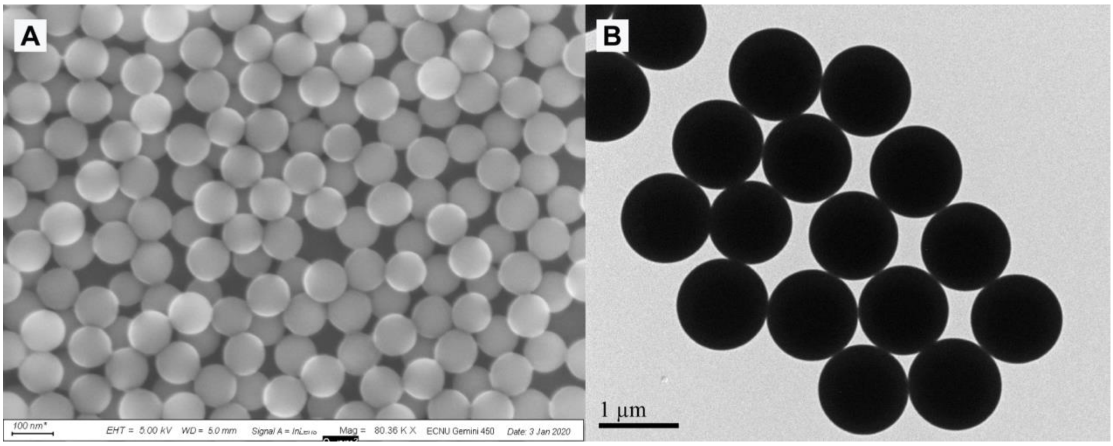

Figure S7. The images of MPs used in this study. (**A**) 0.1 μm-MPs under scanning electronic microscope; (**B**) 1 μm-MPs under transmission electronic microscope.

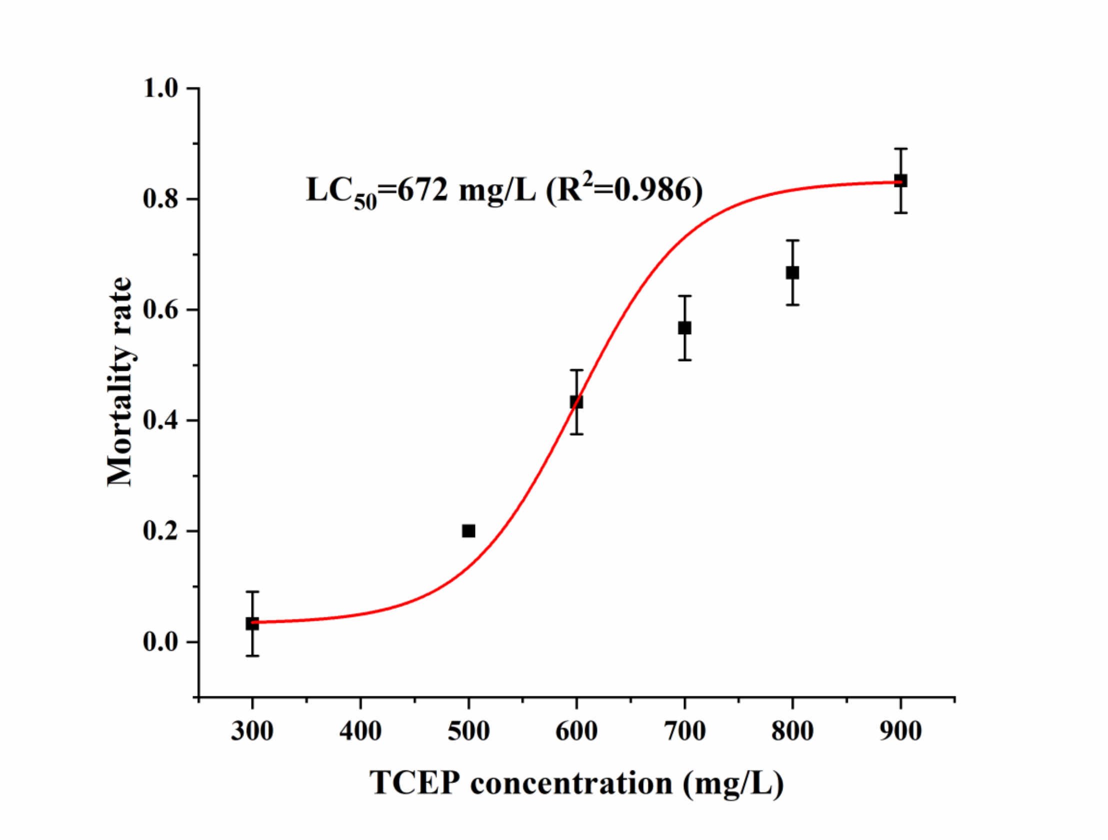

Figure S8. The mortality rate of *B. plicatilis* after 48 h exposure of TCEP.

1. A detailed method for acute toxicity of TCEP on rotifers

The concentrations of TCEP were set as 300, 500, 600, 700, 800 and 900 mg/L, and sterilized seawater without TCEP was used as the control group. For each group, 1 mL of sterile seawater containing a series of TCEP concentrations was injected into one well of a 24-well plate without bait. 10 robust rotifers larvae (<2 h) were selected and added into each well. There were three replicates for each treatment. The condition was similar to those described in section 4.1. The number of surviving adults was recorded every 24 h under a stereomicroscope (XTL-400, Guiguang Instrument Co. LTD, Guilin, China), and the experiment finished after 48h.

Based on the data obtained from experiments, the mortality was calculated and the 48-h LC_{50} value of TCEP for rotifers was obtained by fitting Logic model using Origin 2018 (Origin Lab. Corporation, Northampton, MA, USA) and SPSS 25.0 (SPSS Inc., Chicago, IL, USA).

2. The calculation of population growth-related parameters

Population growth curves were generated using Origin 2019, The following parameters were evaluated using logistic formula (Sigmaplot):

$$Nt = \frac{K}{1 + e^{a-rt}}$$

$$T_P = \frac{a}{r}$$

t: time;
Nt: the population density at time t;
K: the carrying capacity of the environment;
r: the maximum instantaneous growth rate;
a: related to N_0 (the theoretical value of population density at time t=0,);
Tp**:** the growth inflection point.
